# The Medical Schools and Hospitals of Paris

**Published:** 1875-02-15

**Authors:** C. C. Matteson

**Affiliations:** Paris


### THE MEDICAL SCHOOLS AND HOSPITALS OF PARIS.

EDITORS MEDICAL EXAMINER: In these days, when the majority of American medical men look upon Germany as headquarters for European medical affairs, it may not be uninteresting to note that which is being accomplished in Paris. Certainly this city, with its 17,000 hospital beds, can offer a variety of cases that it would be difficult to find elsewhere, and its lack of popularity at present cannot be ascribed to want of material for study. The long distances that separate the various hospitals cause much time to be lost in visiting them, and this is one drawback. Perhaps, as some think, the tendency of the French to consider that out of France little or nothing can be found, has had its inevitable result in permitting more liberal nations to distance them. Be these things as they may, the profession in Paris is doing good work to-day, and the journals are replete with reports of interesting and carefully-studied cases.

During the last annual competitive examination for the vacant places as assistant professor in the Faculty of Paris, the opinions that were advanced by many of the candidates upon the origin of the lymphatic system, led M. Sappey, Professor of Anatomy, to interrupt his regular course of lectures in order to give his views upon the subject in question. He announced his intention to his class in this way: "Far be it from me to deny the value of the young school, but I cannot help showing you that among the statements that have been advanced some great errors have been made which I cannot permit to pass without refuting, considering this course my duty and your right. I wish in my turn to treat of this question. They have advanced German science. I wish to show you French science, and it is by facts that I will answer the assertions made. I will do it courteously, but with entire impartiality, and I hope that from this discussion will come some light."

In his lectures on this subject, he reviewed the history of researches upon the lymphatic system, and showed how the Recklinghausen or "German" investigations were carried on by means of coloring the lymphatic epithelium with silver, while to himself was due the better method of coloring the lymph itself by reactives. From this better method of investigation he was able to arrive at the following conclusions:

1st. The lymphatic system arises by a network of capillicules and of lacunes which contain some simple granulations.

2d. From this network the lymphatic capillaries part, and, at first, are only a series of lacunes.

3d. In uniting, these lymphatic capillaries form the central trunk that is found in all the papilles and villosities.

4th. This central trunk anastomoses with the neighboring central trunks, and from these anastomoses results the plexus that was injected by the method of Fohmann and Panizza.

The course and lectures of M. Sappey have been rather severely criticized by the medical journals, and the general opinion seems to be that he has failed most signally in doing what he promised. The *Progres Medicale* closes an article on this attempt of M. Sappey as follows: "Finally, two of the lectures, given before a large audience, have not yet reversed the facts announced by the young anatomists. Enthusiastic applause has greeted the Professor, whose merit and learning all know, and whose remarkable works all appreciate. But for many this applause is directed toward the force of his convictions and the ability with which he defends them, rather than to their justness."

In another article the same journal states that if M. Sappey will take the trouble to visit the College of France he will there see preparations that demonstrate the facts that he denies. This is rather a severe charge of ignorance as to what is being accomplished by research, especially when emanating from a French journal against a professor at the medical school.

During the past month, Dr. Bouchut has been making a series of experiments with eserine (calabarine) in the treatment of chorea. In his wards at the Hopital dez Enfants Malades, he gave hypodermic injections of one and a quarter millegrammes of the soluble sulphate of eserine to twelve cases of that affection, and, as far as the disease was concerned, with good results. The patients were from seven to thirteen years of age, and in all the chorea was well marked, though not severe. Every morning the dose was repeated, and in four days three of the children were completely relieved from any choreic symptom, in a week three others, and ten days after the first injection, with one exception, all were manifestly improved. The immediate effect of the drug was an intense nausea, followed by vomiting. After some hours there appeared a partial paralysis of the diaphragm, which rarely lasted longer than half an hour. The effect of the eserine upon the pupil was very curious, for in no case did there appear a contraction, but in the majority there was a dilatation of that orifice. The results from this line of treatment are very satisfactory as regards the disease, but, like those from the use of tartar emetic, are too severe upon the patient to find general acceptance.

In the same wards is a very interesting case that illustrates the remarkable tolerance of chloral in children. The patient, a girl of eleven years, was admitted some two months ago for a chorea in its most aggravated form. She was treated with the hydrate of chloral and, for six weeks, was given three grammes (46 grs.) of that remedy night and morning. Of the chorea there were hardly any signs left at the end of that time, and the girl had no derangement of organ or function, nor, indeed, of appetite. While examining this case, Dr. Bouchut stated to me that he had never seen a bad symptom follow from the use of chloral in treating children, and certainly he has had opportunities for studying the effects of that remedy.

Bouchut himself is a close investigator, and the care with which he notes every case, during his ward visits, gives a guarantee as to the correctness of his observations. Though he has been studying the diseases of children for thirty years, his ideas do not seem to have fallen into an unchangeable routine, as is so often the case, but he is always ready and eager to try some new remedy, not contenting himself with the past. His investigations on cerebroscopie are still being carried on, and furnish some valuable results that seem too little noticed. (In some future letter I will give his latest conclusions on this subject.)

Under the title of "Employment of the Cyanides in the Treatment of Acute Articular Rheumatism," Dr. A. Luton, of Reims, publishes an interesting article in the *Bulletin General de Therapeutique*, of January 15th. His experiments have been made especially with the cyanides of zinc and of potassium, and he recommends the former more particularly from its being free from any disagreeable taste or odor. He gives the notes of some ten cases in which he made use of the cyanides, and, were his results, as given, to be had in similar cases, we might, with him, be led to regard a cyanide as "an antiarthritic nearly specific."

The remedy was given in quantities of 5 to 20 centigrammes, during the twenty-four hours, in pills or powder. The conclusions that he has arrived at are very forcibly expressed in his closing sentences: "Finally, it is *certain* that the cyanides cure acute articular rheumatism, in its fundamental form, and in its different varieties. They cure in singularly shortening the course of the disease, and in diminishing the risk of complications that properly belong to the disease. One of the essential conditions of every good anti-rheumatismal medicine is *rapidity*. Add to this that the remedy is not disagreeable to take, and that it is anodyne, and you have the three great recommendations of the school: *cito, tuto et jucunde.*"

Should the cyanides but half respond to such an enthusiastic encomium, the profession has found a powerful means of combating a subtle enemy. C. C. MATTESON, M.D.

PARIS, Jan. 18th, 1875.

